# Treatable brain network biomarkers in children in coma using task and resting-state functional MRI: a case series

**DOI:** 10.3389/fneur.2023.1227195

**Published:** 2023-08-10

**Authors:** Varina L. Boerwinkle, Bethany L. Sussman, Jordan Broman-Fulks, Emilio Garzon-Cediel, Kirsten Gillette, William R. Reuther, Mark S. Scher

**Affiliations:** ^1^Division of Pediatric Neurology, Department of Neurology, University of North Carolina, Chapel Hill, NC, United States; ^2^Neuroscience Research, Barrow Neurological Institute at Phoenix Children's Hospital, Phoenix, AZ, United States; ^3^Division of Pediatric Neurology, Emeritus Scholar Tenured Full Professor Case Western Reserve University School of Medicine Department of Pediatrics, Rainbow Babies and Children's Hospital/University Hospitals Cleveland Medical Center, Cleveland, OH, United States

**Keywords:** coma, resting state functional MRI, pediatric, disorders of consciousness, cognitive motor dissociation, case report, acute brain injury

## Abstract

The withdrawal of life-sustaining therapies is frequently considered for pediatric patients with severe acute brain injuries who are admitted to the intensive care unit. However, it is worth noting that some children with a resultant poor neurological status may ultimately survive and achieve a positive neurological outcome. Evidence suggests that adults with hidden consciousness may have a more favorable prognosis compared to those without it. Currently, no treatable network disorders have been identified in cases of severe acute brain injury, aside from seizures detectable through an electroencephalogram (EEG) and neurostimulation via amantadine. In this report, we present three cases in which multimodal brain network evaluation played a helpful role in patient care. This evaluation encompassed various assessments such as continuous video EEG, visual-evoked potentials, somatosensory-evoked potentials, auditory brainstem-evoked responses, resting-state functional MRI (rs-fMRI), and passive-based and command-based task-based fMRI. It is worth noting that the latter three evaluations are unique as they have not yet been established as part of the standard care protocol for assessing acute brain injuries in children with suppressed consciousness. The first patient underwent serial fMRIs after experiencing a coma induced by trauma. Subsequently, the patient displayed improvement following the administration of antiseizure medication to address abnormal signals. In the second case, a multimodal brain network evaluation uncovered covert consciousness, a previously undetected condition in a pediatric patient with acute brain injury. In both patients, this discovery potentially influenced decisions concerning the withdrawal of life support. Finally, the third patient serves as a comparative control case, demonstrating the absence of detectable networks. Notably, this patient underwent the first fMRI prior to experiencing brain death as a pediatric patient. Consequently, this case series illustrates the clinical feasibility of employing multimodal brain network evaluation in pediatric patients. This approach holds potential for clinical interventions and may significantly enhance prognostic capabilities beyond what can be achieved through standard testing methods alone.

## Introduction

Approximately 3% of all deaths among pediatric intensive care unit (PICU) admissions are attributed to a poor neurological status necessitating the withdrawal of life-sustaining therapies (WLSTs) (1–3) However, it is important to consider that a subset of these patients not only survive with positive outcomes but may also exhibit covert consciousness, referred to as cognitive motor dissociation (CMD), during the WLST-consideration process, creating an ethical dilemma (4). CMD, which is associated with improved outcomes, has been observed in 15% of adult cases with acute severe disorders of consciousness (DoC) but has yet to be reported in children with acute coma (5). The recent Pediatric Acute Coma Care and Research Meeting, supported by the Neurocritical Care Society (NCS) and the National Institute of Neurological Disease and Stroke (NINDS), recently emphasized the significance of this topic (6).

Advancements in multimodal brain network evaluation, such as functional MRI, have the potential to revolutionize the field for children. This includes the detection of CMD in children and the identification of treatable network disorders across all age groups, as suggested by various professional society guidelines (7, 8). However, the practical utility of multimodal brain network evaluations for clinically relevant CMD diagnosis and the identification of treatable network disorders within a feasible timeframe remain largely unexplored (9). At present, there are no established treatments for network disorders in cases of acute disorders of consciousness (DoC), beyond seizures detectable by EEG and neurostimulant therapy, regardless of age (10).

In this report, we present a preliminary case series from our clinical experiences with multimodal brain network evaluation for demonstrative purposes. The primary objectives were to identify treatable pathological networks, previously observed in pediatric epilepsy and neonatal acute brain injury cases (11–15), detect CMD, and provide prognostic information during the clinical progress of PICU patients. The results of the multimodal brain network evaluation directly influenced patient care, potentially leading to the return of clinical consciousness as observed by examinations and the avoidance of WLST.

## Materials and methods

The clinical case series involving three patients conducted between April 2018 and April 2021 was determined by the local institutional review board not to constitute human subject research. During the acute care period, the families provided consent for the publication of the case series, and this consent was duly witnessed and documented in the medical records. Subsequently, in accordance with the guidelines set forth by the publisher for case series, the families also provided written consent in the specified format for publication in the journal.

Furthermore, the parents of patients 1 and 2, as well as the assenting patients themselves, granted consent for the citation of their family interviews, which were published during the NCS World Coma Day Event (1, 2). The consent explicitly acknowledged the utilization of video and the inclusion of specific content related to patient 1. The cases selected for inclusion in this study were chosen to exemplify the potential of coma biomarkers in children. The Supplementary material contains de-identified medical care data related to the case series.

The objective was to characterize different subtypes of acute disorders of consciousness by identifying patients with preserved neural networks who exhibited an inability to respond volitionally during examinations and to detect potentially treatable network disorders. The decision to conduct multimodal brain network evaluations, including anatomical magnetic resonance imaging (MRI), resting-state functional MRI (rs-fMRI), task-based fMRI (task-fMRI), electroencephalogram (EEG), examinations, and other appropriate modalities and to incorporate the results into clinical care was initially made by the institution's leadership, which included those from the intensive care unit, neurology, and neurocritical care. The purpose was to utilize these tests to guide patient management.

Rs-fMRI scans were performed in cases where there was a clinical indication and when it was deemed safe for the patient to be transported and undergo the scan without sedation. Anatomical MRIs were conducted alongside the fMRI scans as part of the indicated evaluation integrated into the patient's care plan. Parents provided consent for the clinical rs-fMRI and task-fMRI, as they would for any clinical tests, and these tests were not conducted under a research protocol.

The clinical integration of rs-fMRI was initiated in 2012 at Texas Children's Hospital. Since then, the practice has undergone review by the American Medical Association (AMA) and the Federal Drug Administration (FDA), both of which have determined that it complies with their respective regulations. According to the AMA's guidance, the professional interpretation of rs-fMRI is billed to payors using the current procedural technology (CPT) code 76498. The FDA has concluded that the practice described here does not involve the sale of software as a medical device, and as such, it falls outside the scope of FDA regulation as the FDA does not govern medical practice.

Therefore, the medical practice of integrating professionally interpreted rs-fMRI, analyzed using in-house software, is deemed compliant with FDA regulations. The application of this technology in other institutions is progressing in accordance with guidelines set forth by professional societies, and recommendations have been published in this regard (8).

The multimodal brain network evaluations comprised various passive tests that were applied as they became available and were clinically indicated. These tests included rs-fMRI, continuous video EEG (cvEEG), passive brain network activation testing with visual-evoked responses (VEP), somatosensory-evoked responses (SSEP), auditory brainstem-evoked responses (ABRs), selective passive task-fMRI (such as the examiner moving the patient's hand), CvEEG reactivity to environmental stimulation, and volitional-activated brain network testing through task-fMRI.

Comparatively, task-fMRI was not available clinically for patient 2 and so was not performed.

### ICU care and diagnostics

#### Acute-period diagnoses

The pediatric Glasgow Coma Scale (pGCS) is used to assess the level of mental status and the presence of a disorder of consciousness (DoC) (16, 17). From retrospective medical record review, the pGCS scores were extracted in preference to using care provider documented scores, and if scores were not documented on a given day, then the documented exam components corresponding to the scale were utilized.

#### Neurological and consciousness examinations

Neurological and consciousness examinations were conducted upon admission and subsequently as necessary. These evaluations were performed by the neurocritical care team. Any subsequent examinations and new events, such as the onset of new weakness, worsened encephalopathy, concern for seizure activity, or new abnormal EEG findings, were thoroughly documented. Additionally, repeat insults to the brain were recorded to differentiate outcomes resulting from acute-period examinations from those associated with additional brain insults.

#### Electroencephalogram

CvEEG was conducted throughout the acute brain insult period as required, typically prior to MRI scans. The initiation of cvEEGs usually occurs within 0–24 h of admission or upon the diagnosis of a suspected brain insult, depending on the clinician's judgment regarding indications of potential seizures or the need for assistance in determining adjustments to antiseizure treatment. Seizures were defined as rhythmic discharges that evolve in frequency, amplitude, morphology, and/or spatial distribution, lasting for at least 10 s. CvEEG readings encompassed various findings, including (1) normal, (18) mild, indicating interpretations suggesting dysmaturity such as mild discontinuity, (2) seizure, and (11) flat, denoting cvEEG background suppression and severe discontinuity. Standard 19-lead EEG arrays were used, and cvEEG monitoring was employed. The Natus XLTEK System EEG/Sleep Acquisition with Neuroworks and Sleepworks software version 8.5 was utilized for EEG recording, operating at a sampling rate of 200 Hz and employing a standard EEG montage (19). The brain monitor amplifier, along with the “Connex” breakout box, was used as the EEG/sleep amplifier.

#### MRI conditions and timing

In order to undergo a clinical MRI, all patients were in a safe and stable condition for transportation to the scanner and received continuous monitoring from the respective hospital care teams. The total scan duration was ~1 h. During the scan, patients received analgesia or conscious sedation-level anesthetic agents as part of their care plans. In these cases, sedation was required to minimize patient movement during the imaging process. Patient 1 received two separate fMRI scanning sessions, which were pre- and post-antiseizure medication. Patients 2 and 3 had only one rs-fMRI during the acute period.

#### Anatomical MRI

All sequences were in a 3 Tesla MRI (Ingenuity, Philips Medical Systems, Best, Netherlands) equipped with a 32-channel head coil. Diffusion-weighted imaging (DWI) sequences with corresponding apparent diffusion maps were acquired. The parameters for the DWI sequences were as follows: 2D imaging, 4 mm slice thickness, TR of 4.9 msec, TE of 0.071 msec, 5 mm spacing between slices, 124 phase encoding steps, percent phase field of view of 96, flip angle of 90, and field of view (FOV) of 250x250 mm. Additionally, a T1-weighted 3D whole-brain sequence was obtained for anatomical reference, with a TR of 2.5 ms, a TE of 331 ms, a flip angle of 90, a slice thickness of 0.9 mm, and an in-plane resolution of 0.8 × 0.8 mm. The results of the scans were interpreted by a neuroradiologist.

#### Task-fMRI

The task-fMRI consisted of two runs, each containing five blocks lasting 24 s, separated by 24-s interstimulus intervals. The patient wore headphones during both runs, through which tasks were administered by a technician. In the first task run, the patient was instructed to move their arms. In CMD, a positive task-fMRI with this motor command would indicate activation of the primary sensory-motor cortex. In the second task run, the patient was instructed to continuously repeat the phrase “cat, dog, mouse” during the activation periods. In CMD, the expected finding would be the activation of the regions associated with language reception, including the superior temporal gyrus, language repetition, including the primary sensory-motor mouth region, and language expression, including the inferior frontal gyrus.

Regarding task-fMRI, in the case of patient 1, this test was performed to evaluate for CMD and involved commands for leg movement and subvocalization. During the second MRI scan, because we saw positive activation during the commands for arm movement and word repetition, we also performed an active listening task of greater difficulty, which requested that he distinguish animal words from a list of age-appropriate non-animal words by repeating only the animal words. Comparatively, task-fMRI for CMD was not clinically available during the time period of care for patient 2; therefore, it was not performed.

#### Rs-fMRI methods

a. Acquisition

T2-weighted images were acquired with a TR 2 s, TE 30 ms, matrix size of 80 × 80, flip angle of 80 degrees, number of slices to cover at least the supratentorium, slice thickness such that the voxel size is 3.4 × 3.4 × 3.4 mm^3^, no slice gap, and inter-leaved top-down acquisition. The number of total volumes was 600, split between two equally timed runs of each ~10 min.

b. Preprocessing

Standard rs-fMRI preprocessing steps were implemented, including the removal of non-brain structures, deletion of the first five volumes to mitigate T1 saturation effects, high-pass filtering at 100 s, inter-leaved slice time correction, minimal spatial smoothing at 1 mm, and motion correction using MCFLIRT (11, 20). MCFLIRT is an automated motion correction tool designed for fMRI time series that uses the brain image taken from the middle of the rs-fMRI run to transform the other images to be in line using trilinear interpolation. In the majority of cases where the subject motion is minimal, this scheme is geared to improve the accuracy of the final images. In our study, all subjects exhibited motion-induced displacement of < 1.0 mm in any direction, though it is possible for motion correction to induce differences in the data if the motion was beyond this limit. This is important because, in precision medicine, statistical verification is substantially less robust than what can be achieved in groupwise research studies with higher statistical verification. Individual functional scans were registered to the patient's high-resolution anatomical scan using linear registration (11, 20), with optimization achieved through boundary-based registration (21).

c. Analysis

Independent component analysis (ICA) was employed to analyze the pre-processed rs-fMRI signal and partition it into oscillating subsignals (22). These subsignals, referred to as independent components (ICs), can originate from brain networks or sources of noise, such as the MRI scanner, respiration, cardiovascular activity, and cerebrospinal fluid pulsation. Differentiating between noise and brain networks presents a challenging task, as evidenced in the relevant literature (11, 13, 23–26).

Each brain network exhibits distinctive oscillation patterns in terms of its oxygen concentration, distinct from other brain networks. This characteristic facilitates the identification of individual brain networks. In functional gray matter, the normal time course of this oscillation pattern is typically smooth, slow, and generally below 2 Hz. Conversely, abnormal brain networks often display fast and erratic blood oxygen level-dependent (BOLD) time courses (11).

A recent meta-analysis based on rs-fMRI ICA and principal component analysis provided supporting evidence from multiple investigators' ability to classify the ICA subsignals with meaningful results (27). Automated rs-fMRI interpretation offers an alternative to the manual classification of independent components (ICs); however, this has not been validated in acute brain injury (28). Similar to the interpretation of anatomical MRIs, the interpretation of rs-fMRI still relies on individuals with clinical expertise, making it susceptible to the same levels and types of subjective bias (29, 30).

The application of this approach has been extensively documented in pediatric clinical settings (11–13, 15, 31–35). It has been previously employed in a population of pediatric patients with intractable epilepsy, primarily resulting from prior brain injuries and congenital causes. Moreover, subsequent repeat studies in the same individuals have shown correlations with changes in their clinical condition, thereby enhancing the validation of its individualized application (13).

MELODIC (22), a freely available ICA software, was used for this study. Bayesian computation was utilized to determine the total number of detected independent components (ICs) for each patient. Consistent with prior studies (10, 28), the standard local false discovery rate was employed to establish an IC threshold of *P* < 0.05. To differentiate between noise components and neuronal signals from established resting-state networks, expert personnel conducted manual assessments based on spatial patterns (11) and a BOLD frequency < 10 Hz/100 (11, 13).

The classification of networks as normal, atypical, or undetected was based on expert interpretations documented in the clinical rs-fMRI reports. The evaluated resting-state networks (RSNs) in this study included the motor, frontal, parietal, vision, temporal, basal ganglia (BG), language/frontoparietal (Lang/FP), and default mode networks (DMNs). Additionally, connectivity indicative of seizure onset zones (rs-SOZs) was assessed, as discussed below.

#### Rs-SOZ identification

Through meta-analysis, rs-SOZ has been observed in drug-resistant epilepsy cases, where epileptogenic networks distinguish those with abnormal findings from normal individuals (27). The presence of rs-SOZ correlates with the localization of seizure onset zones (SOZs) determined through intracranial EEG, is associated with surgical outcomes in epilepsy patients (11), and normalizes in conjunction with network-targeted therapy and 1-year seizure outcomes (13). In this study, the EEG results were not used to identify rs-SOZ. The prior study, which evaluated intracranial EEG SOZ against rs-SOZ, also did not use the intracranial EEG to identify the rs-SOZ but rather retrospectively evaluated for spatial concordance as a validation effort.

**Figure 1:**
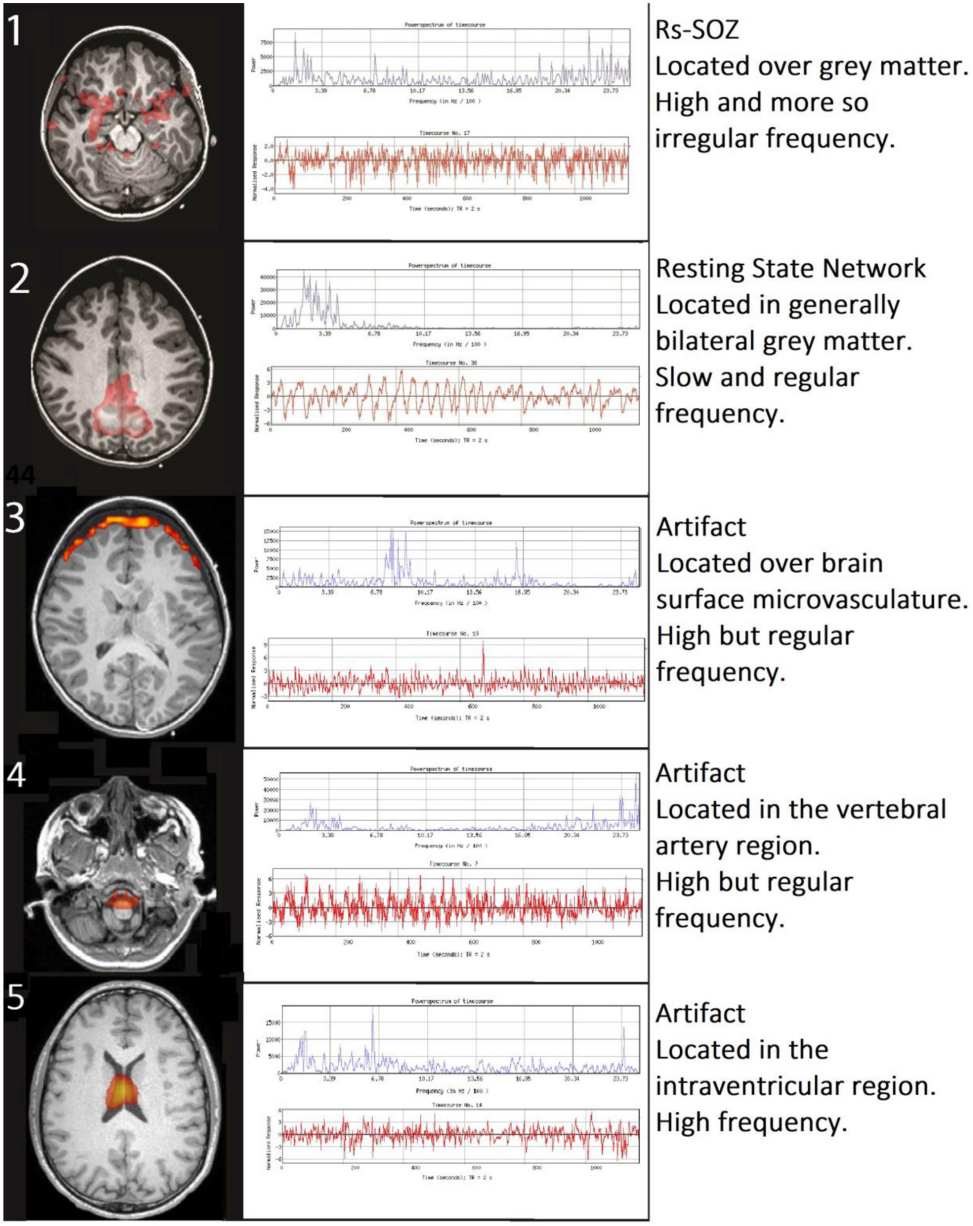
Differentiating rs-SOZ from resting state networks and artifacts. The generally detected spatial and temporal features of rs-SOZ are shown in row 1. Comparatively, row 2 has an example of a resting state network, and examples of rs-fMRI artifacts are in rows 3–5, with corresponding text describing typical differentiating features.

Rs-SOZ is distinguished from RSN and artifacts by differentiating spatial and temporal characteristics (Figure 1). Namely, rs-SOZ is largely located over gray matter, though they may have a tail toward the central structures. Their time courses have irregular and erratic changes in frequency, compared to RSN that are generally more regular in time course throughout. Finally, the power spectrum of rs-SOZ frequencies is shifted toward higher frequencies, generally above 6.7 Hz/100, which is above normal. In comparison, noise from artifactual sources has highly identifiable traits including a spatial location largely over the ventricles due to cerebrospinal fluid (CSF) pulsation, a ring around the outside of the brain due to either CSF pulsation or vascular sources, or a location over the known cerebrovascular structures. The noise-related signal source time courses have more uniformly regular frequency generally than rs-SOZ, but the power spectra are shifted higher than normal. However, a key difference in the abnormally high-power spectrum of rs-SOZ and noise is that rs-SOZ will have a more diffuse spectrum of frequencies, while noise will generally contain a prominent narrow band of frequencies. This is likely due to the regular frequency of the pulsatile nature of the cardio-respiratory cycle in brain physiology, which is largely non-neuronal in these spaces that are not within brain tissue.

Considering the heightened risk of subclinical seizures during the acute brain injury (ABI) phase and the potential for false negatives in EEG readings due to insufficient event-triggered vigilance or deep-seated seizures that are challenging to detect on surface EEG, there is a need for a data-driven, objective biomarker that can identify treatable network disruptions. This objective could be pivotal in reducing inappropriate WLST in cases of subclinical seizures occurring during ABI. Additionally, given the connection between rs-SOZ and epileptogenic sources in epilepsy, the risk–benefit ratio was interpreted as positive by the clinicians who integrated it into the care of the patients in this case series by providing antiseizure medication if rs-SOZ was detected.

## Results

**Figure 2:**
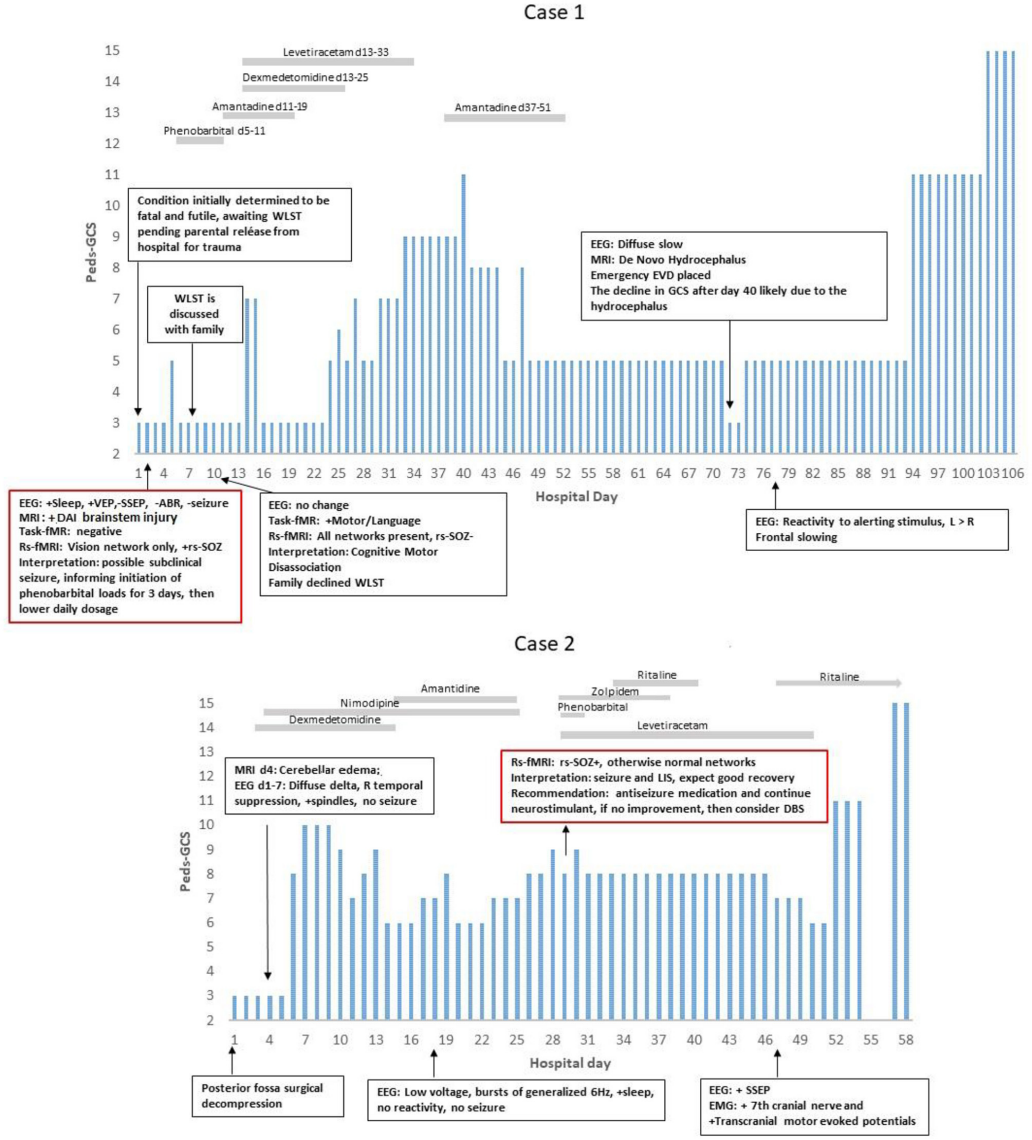
PEDS-GCS and hospital day. In Case 1, the rs-fMRI most networks were not detected, and seizure networks (rs-SOZ+) were found, yet the EEG was without a seizure. After antiseizure treatment, the rs-SOZ resolved, and the resting state networks were normalized. The task-fMRI, which was initially negative, then showed command followed by network activation. Thus, he was determined to have cognitive motor dissociation (1). In Case 2, rs-SOZ was also positive, the EEG was without a seizure, and antiseizure medication was then given. However, this rs-fMRI did show networks that were otherwise normal, raising the concern for either cognitive motor dissociation or locked-in syndrome. At that time, he had facial movements of repetitive stereotyped grimacing that occurred with and without stimulus and were difficult to interpret, especially in relation to painful stimuli and prompts for commands. The task-fMRI service for DoC was not available. After recovery, the patient described being aware while in the ICU and unable to communicate. He said, “It was boring. I could see people walking in the hallway. When doctors came in the room to talk, then it was more interesting. I played choose your own adventure in my mind (2)”.

**Figure 3:**
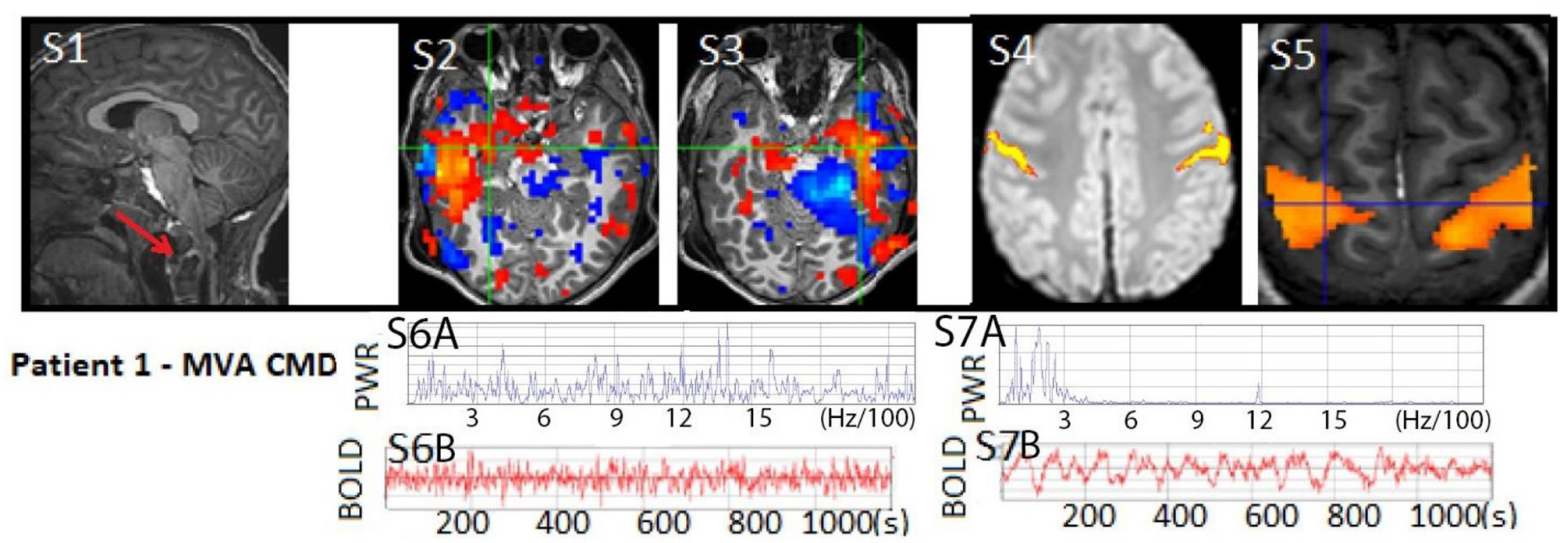
Patient 1: (S1) T1W MRI with cervical level one atlanto-occipital dislocation (RED arrow) and same level spinal cord displacement with edema indicating severe spinal cord injury, often considered a fatal injury, not shown (18). Anatomical MRI otherwise showed patchy diffuse signs of diffuse axonal injury. (S2–S3) rs-SOZ with a red-blue activation–deactivation pa**t**tern and (S6A–B) fast frequencies in the BOLD power spectrum and time course. (S4–S5) Post-seizure treatment task-fMRI motor activation and normalized rs-fMRI motor network with (S7A–B) normal BOLD power spectrum and time course. The patient's motion during rs-fMRI was < 0.2 mm in any direction.

**Figure 4:**
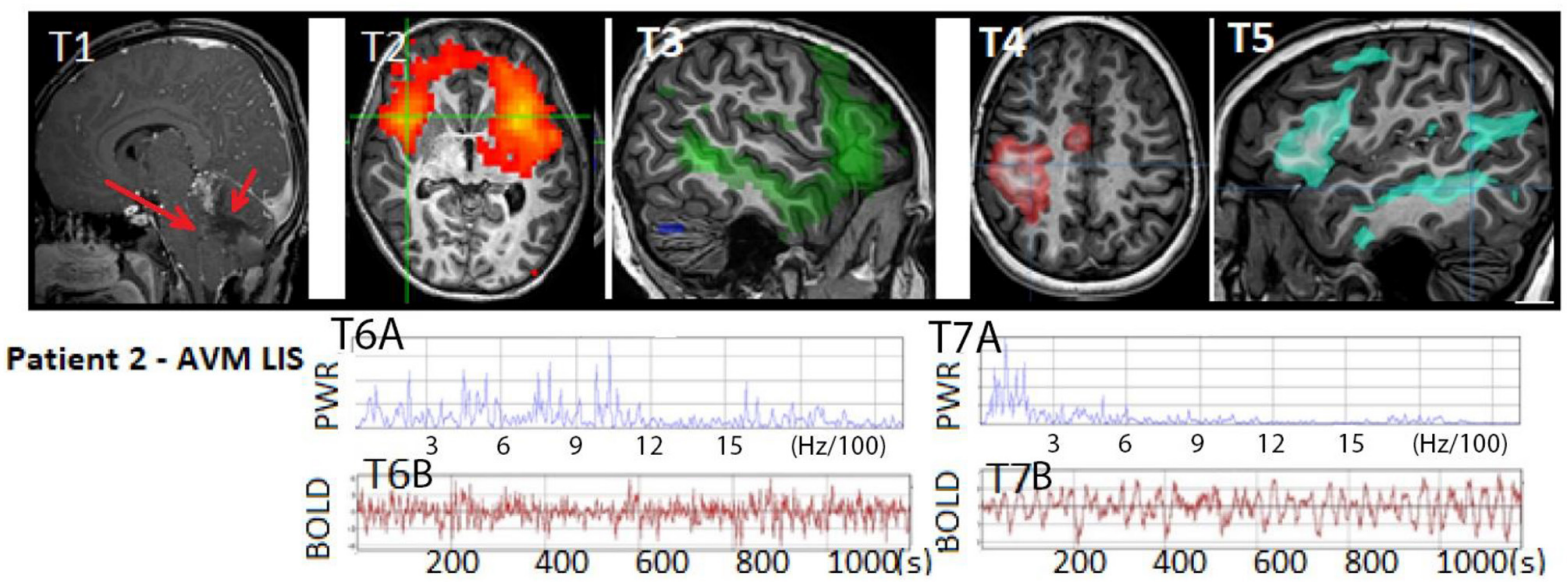
Patient 2: (T1) T1W MRI AVM hemorrhage (RED arrow on right) within the cerebellum with resultant mass effect on the brainstem and anterior cerebellum (RED arrow on left) indicative of life-threatening injury and an indication for emergent posterior fossa craniectomy, which occurred after this image was acquired. Anatomical MRI otherwise showed patchy diffusion restriction in the brainstem, tegmentum, and cerebellum (not shown). (T2) rs-SOZ with (T6A–B) fast frequency in the BOLD power spectrum and time course. (T3–T5) Normal resting state networks of language, sensory-motor hand, and frontoparietal networks, with (T7A–B) normal BOLD power spectrum and time course. Images above are representative of the findings. Categorization of rs-fMRI networks in children with and without epilepsy is described (11). Normal neuronal BOLD has an oscillatory pattern that has <6 Hz/100, whereas cerebrospinal fluid pulsation-related noise has a faster regular cadence, and rs-SOZ has > 6 Hz/100. Artifacts have a spatial distribution that may not respect brain boundaries. Rs-SOZ is spatially largely over gray matter. The patient's motion during rs-fMRI was < 1 mm in any direction.

**Figure 5:**
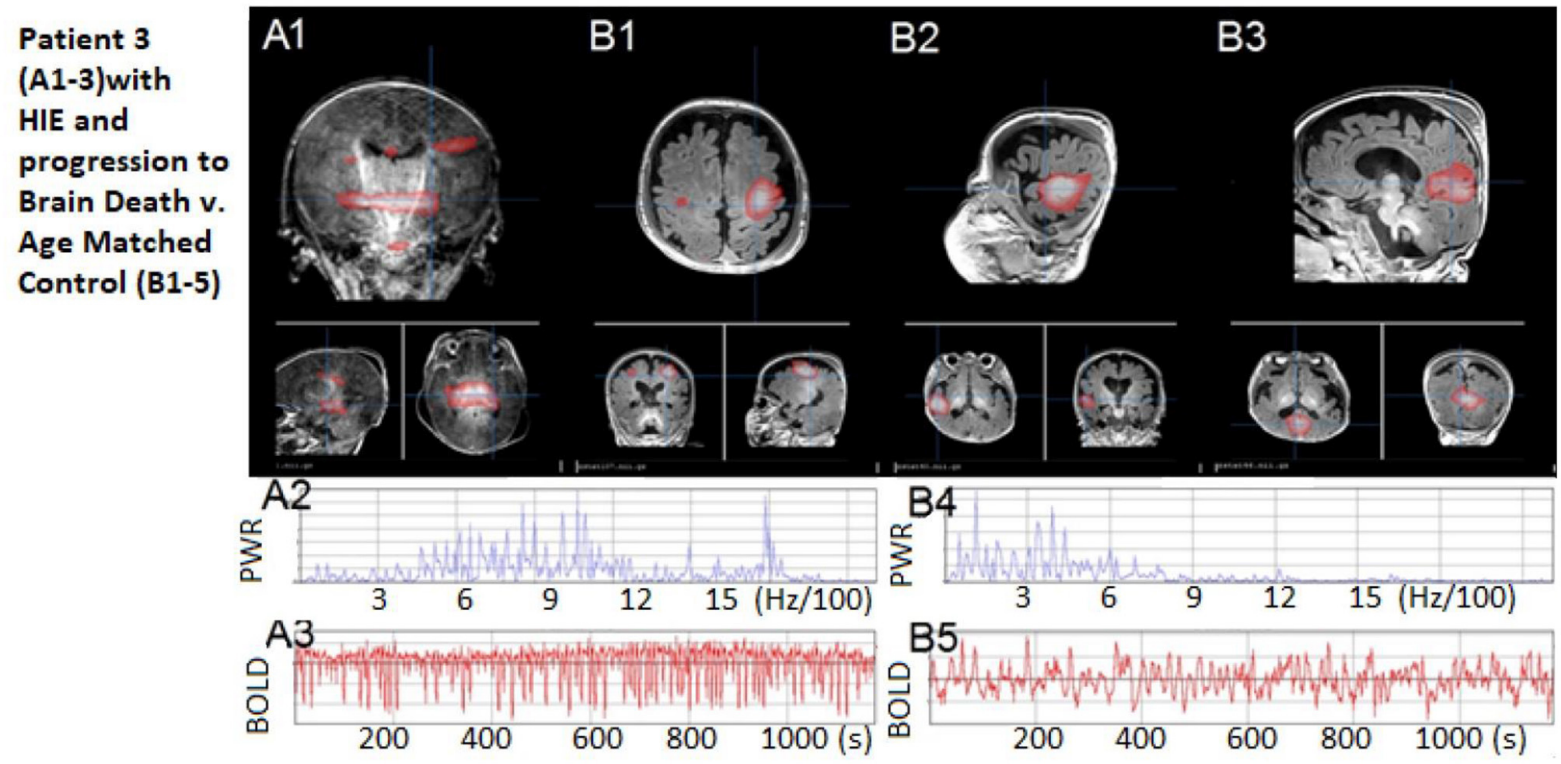
Patient 3: Patient 3: (A1) No rs-fMRI networks, only artifact signal with (A2–A3) high-frequency power spectrum and regular cerebral spinal fluid-pulsation artifact time course, as expected in an infant who progressed to brain death shortly afterward. (B1–B3) Comparisons of neonate with normal resting state networks – shown are sensory-motor, language, and default modes, respectively. (B4–B5) A representative BOLD time course and normal power spectrum from the normal neonate's networks. The patient's motion during rs-fMRI was < 0.2 mm in any direction.

Timelines depicting pGCS, treatment, and examination changes (Figure 2) and expanded views of MRI (Figures 3–5) highlight the clinical usefulness of multimodal brain network evaluations. A summary of EEG and rs-SOZ results is provided in Table 1. Additional details are shown in Supplementary material.

**Table 1:** Summary of EEG and rs-SOZ results.

**Patient**	**EEG results**	**Rs-SOZ result**
1	Days 1–10: no seizure, no epileptiform activity, +sleep, +VEP, -SSEP, -ABR	+rs-SOZ in right and left medial and anterior temporal regions
2	Days 1–7: no seizure, no epileptiform activity, diffuse delta, right temporal suppression, +spindles	+rs-SOZ in inferior and basal prefrontal and temporal regions
3	Days 1–4: no seizure, no epileptiform activity, diffuse delta	-rs-SOZ

### Case 1

A 6-year-old boy, previously experiencing normal development and without any significant medical, family, or social history, suffered severe traumatic brain injury (TBI), diffuse axonal injury (DAI), and atlanto-occipital dislocation with accompanying severe cervical (C1)-level spinal cord and bulbo-medullary injury (as depicted in Figures 2, 3), which is often considered fatal (18). Furthermore, the patient experienced cardiac arrest. Upon admission, he was in a state of acute flaccid coma with closed eyes and showed no response to stimuli, exhibiting only sluggish reactivity in one pupil. This condition was observed on the first day of hospitalization. Although the initial prognosis indicated a futile and terminal state, for which it was expected that he may progress to brain death, the decision to continue life support was made as the parents were undergoing their hospitalization. Consequently, a comprehensive assessment of multimodal brain networks was conducted, leading to therapy and further testing. Miraculously, the patient survived and now possesses communication and cognitive abilities comparable to his chronological age, despite being quadriplegic.

On day 2 (d2), antiseizure medication treatment was initiated based on rs-fMRI findings, which detected rs-SOZ suggestive of epileptogenic activity in deep brain regions that were not observed on cvEEG monitoring conducted during that period. Task-fMRI performed on d2 showed no reactivity initially but demonstrated activation in response to commands on d10, indicating command-following (CMD), despite the patient being in a coma according to bedside examinations. Consequently, network improvement was first detected through rs-fMRI and task-fMRI, a significant 17 days prior to the patient regaining consciousness, which became evident on day 27 when he exhibited facial and eye movements in response to commands, despite his quadriplegia. Before d10, the option of WLST was presented to the family, but they declined following the discovery of CMD, as described in a recently published interview (1).

During further hospital care, amantadine was administered from d11 to d19. However, it was discontinued due to family concerns regarding its unclear benefits, particularly considering the patient's covert consciousness. Unfortunately, on d72, newly developing hydrocephalus was identified and likely contributed to deterioration in consciousness. The patient experienced subsequent recovery following the emergent placement of an external ventricular drain (EVD). Later, at 8 years of age, the patient is attending grade-appropriate academic classes, able to communicate orally, and has intermittent recovery of urinary continence. However, there has been only mild improvement in quadriplegia.

The primary conclusion drawn from this case is that rs-fMRI can detect potential epileptogenic signals that may be suppressing brain network activity. Following treatment and repeated testing, confirmed command-following (CMD) can be identified, as initially suggested by rs-fMRI and confirmed by task-fMRI. These findings have significant implications for the consideration of WLST, representing unique advancements in the field of pediatrics with ABI.

### Case 2

An 11-year-old boy with a normal developmental history and no significant medical, social, or genetic background experienced a spontaneous rupture of a cerebellar vermian arteriovenous malformation (AVM) (Figure 4). This event resulted in loss of consciousness and brainstem reflexes, accompanied by cardiac rhythm instability. The patient underwent emergent posterior fossa decompression. Following the procedure, he exhibited fluctuating minimal reactivity for several weeks, meeting the criteria for the unresponsive wakefulness state (UWS). Although he could open his eyes, he lacked target fixation, tracking abilities, and command-following prior to the rs-fMRI evaluation. However, after undergoing a comprehensive multimodal brain network assessment, the patient survived with age-appropriate cognitive abilities and achieved independence in activities of daily living.

On the 19th day, the rs-fMRI revealed signs of potential for consciousness with intact networks, surpassing what would be expected based solely on anatomical MRI findings, which showed patchy diffuse diffusion restriction in the brainstem, tegmentum, and cerebellar regions. Additionally, the patient displayed rs-SOZ activity. His cvEEG did have initial seizure activity in the first 1–3 days, which was treated and completely resolved. Thus, during the subsequent time period leading up to and after the rs-fMRI, his EEG remained negative for seizure. The rs-SOZ was treated with antiseizure medication starting on the 30th day. However, improvement in the patient's condition was delayed, and subsequent fMRI evaluations were not integrated into his care plan. As a result, the response to treatment in relation to the resolution of rs-SOZ during the early phase remains unknown, although the resolution was observed in a later remote rs-fMRI conducted months later.

Ten days after commencing antiseizure treatment, the patient's examination showed improvements in the pattern of eye-opening (day 40), simple command-following (day 47), and full consciousness (day 57). Throughout this period, the patient received multiple neuromodulating medications for various reasons, including amantadine from d15 to d25 (which was discontinued due to the need to rule out sepsis and unclear benefit), zolpidem from d29 to d38 (stopped due to decreased eye-opening), and methylphenidate from d40 to d43 and continued beyond (with possible benefits). After 2 years, the patient is excelling academically and capable of independent functioning in daily activities.

At the age of 15, as shown in his interview, the patient recalls being unable to respond while being aware of the situation in the PICU. This experience aligns with covert consciousness or possibly a form of locked-in syndrome (LIS) (2, 36). During the earlier stages of his treatment when his condition showed limited improvement, some healthcare providers contemplated the option of WLST. However, the results of the rs-fMRI had a significant impact on certain providers, leading them to refrain from recommending WLST to the family. According to the mother's account, the rs-fMRI findings were the most influential indication that the patient had the potential for significant improvement. These results were particularly valuable during a period when other modalities did not provide clear indications of improvement.

This case is notable as it is the first reported instance of a child receiving rs-fMRI for clinical purposes during the acute phase of severe brain injury, which has had an impact on the patient's care. Furthermore, the child himself reports covert consciousness. In conclusion, this case reaffirms the potential of rs-fMRI in detecting potentially epileptogenic signals. However, it also emphasizes the importance of serial imaging for confirming and guiding the course of treatment.

### Case 3

Case 3 presents a contrasting scenario where survival was not achieved. The patient was a 2-month-old boy born at an early-term 37 weeks' diamniotic gestation. Prior to the incident, he was in good health at home with no significant social or family history. However, he was found apneic and unresponsive in the prone position after being fed and placed on his side for sleep. Cardiopulmonary resuscitation was administered until emergency medical services arrived. Initially asystolic, his cardiac activity was restored after receiving two doses of epinephrine within a span of 10 min. Subsequently, the patient underwent multimodal brain network evaluation but unfortunately progressed to brain death. The coroner's autopsy indicated an asphyxiating sleep environment as the cause.

Throughout his stay, the cvEEG showed diffuse low amplitude slowing without a seizure. On the first day, the patient's anatomical MRI was severely affected by movement artifacts, and purposeful-appearing movements were observed (Figure 5). This raised concerns regarding the possibility of a less severe injury that required further evaluation. The medical team felt that the available information at that time was insufficient. Consequently, a repeat MRI with rs-fMRI was performed on the third day to assess the extent of ischemia and ongoing brain network activity, aiming to clarify the capacity for purposeful movement.

The rs-fMRI results revealed no detectable resting-state network or rs-SOZ, indicating incompatibility with the potential for CMD, volitional movement, or consciousness recovery. This pointed to an extremely poor outcome with no evidence of a treatable network disorder. The option of WLST was discussed with the family both before and after consistently unfavorable rs-fMRI findings. The patient passed away due to brain death 1 day later.

This case is noteworthy as it represents the first instance of rs-fMRI being conducted in a child just prior to brain death. In conclusion, this case demonstrates a distinct contrast in findings, with poor network detection compared to the two previous cases, thereby highlighting how rs-fMRI performance may align with the outcome of impending poor outcomes such as brain death.

## Discussion

Within the components of multimodal brain network evaluations, both rs-fMRI and task-fMRI have demonstrated potential for clinical feasibility and value. They have the potential for contributing to the detection of CMD through the screening process of rs-fMRI, followed by task-fMRI confirmation. These evaluations have influenced clinical decision-making, such as avoiding WLST, indicating the need for antiseizure medication treatment, and aiding clinicians in distinguishing between favorable and unfavorable outcomes. These benefits are not achievable through standard tests or the bedside neurological examination alone.

Differentiating between various DoC along the spectrum from CMD to brain death after ABI is crucial for accurate diagnosis. Conditions such as covert consciousness, locked-in syndrome, minimally conscious state, and subclinical seizures, which can mimic irreversible unconsciousness, need to be identified to determine the potential for treatable or recoverable disorders. Therefore, a biomarker's utility across a wide range of findings and outcomes becomes more significant. It is worth noting that the rs-fMRI network biomarker's performance in a case with a poor outcome, like patient 3 who exhibited no detectable networks, serves as a worst-case-scenario control and provides a contrasting demonstration compared to patients 1 and 2. Due to the immaturity of the developing brain in expressing consciousness through examination, the likelihood of misdiagnosis in children and neonates may be even higher than the reported 49% misdiagnosis rate in adults with DoC (14, 37). In cases similar to patients 2 and 3, where individuals exhibit unresponsiveness alongside spontaneous and/or stimulus-induced movements, interpreting these movements as purposeful, seizure activity, or non-epileptic reflexes/automatisms becomes challenging. It is important to note that these paroxysmal events, occurring in 20–50% of patients after brain death (4), contribute significantly to misdiagnosis, particularly within the pediatric population (4, 38).

This diagnostic challenge highlights the relevance of serial multimodal brain network evaluations involving rs-fMRI and task-fMRI studies. In Case 1, task-fMRI successfully identified CMD in a 6-year-old child who had experienced acute brain injury and was in a coma. We anticipate that task-fMRI would exhibit similar performance in children who previously demonstrated the ability to follow auditory commands without explicit prompting but currently cannot express themselves voluntarily due to ongoing motor impairment. Furthermore, classifications of DoC can be achieved by assessing abnormalities in rs-fMRI network patterns (39). Patient 2's recollection of preserved consciousness while in the ICU aligns with the presence of intact resting-state networks, indicating covert consciousness or a form of locked-in syndrome. These positive findings support the integration of results into clinical care, including communication with the family, consideration of low-risk therapeutic interventions, and the avoidance of early WLST.

In terms of prognostication, rs-fMRI provides a data-driven approach to evaluate the presence and extent of normalcy in the brain's networks. The clinical feasibility of rs-fMRI in individuals with acute DoC is enhanced by the fact that it does not require network activation in response to stimuli, the ability to follow commands, or specialized MRI equipment, staff, or scanners (beyond 3T MRI). In the cases discussed here, rs-fMRI results were consistent with the functional recovery of network-dependent processes experienced by patients 1 and 2, or the lack thereof observed in patient 3. These findings align with previous studies that have shown associations between rs-fMRI in neonates with ABI and outcomes related to cognition, motor function, epilepsy, and mortality at both 6 months and 2 years (40). Prospective research validating these findings would enhance confidence in the application of rs-fMRI for prognostication.

In terms of negative test findings, there is still limited understanding in children as the minimal level of brain network function necessary to predict meaningful neurological recovery (37, 41) may be determined by rs-fMRI. This threshold can help guide decisions regarding WLST based on Shewmon et al. (42) study. Despite the risks of delayed apoptotic and autophagic programmed cell death, neuronal viability below this minimum threshold may still be preserved. An adult case of brain death, as presented by Boly et al. (43), provides an example of preserved local connectivity in the posterior cingulate, while lacking long-range connectivity in the default mode network. Additionally, in adults with acute-to-chronic phases of brain injury resulting from anoxia, trauma, or cardiac arrest, connectivity in regions associated with the default mode network has shown predictive value for the recovery of consciousness (4, 44–46). This network-centric approach to detecting minimal brain network activity in cases of severe brain injury has the potential to recalibrate intervention strategies for intensivists. It can inform clinical decisions regarding WLST and facilitate prognostic discussions with families concerning the possibility of survival with recovery potential.

In the context of accurate determinations for WLST in pediatric cases following ABI, it is crucial to consider network-based biomarkers that are relevant to children. Unlike adults, when evaluating the default mode network in early-age pediatric ABI, an important distinction arises due to significant maturational changes occurring in long-range connectivity during this developmental period (47, 48). Consequently, a whole-brain connectivity evaluation, encompassing the whole brain rather than focusing on specific region-to-region connections, becomes more reliable (49). For infants and young children experiencing ABI and DoC, a data-driven approach that considers the whole-brain network pattern is likely to yield better results. This approach is particularly important in avoiding falsely negative findings as selective short- and long-range connectivity may not yet be fully developed in this age group but may still be preserved (47, 48).

Rs-fMRI has the potential to identify deep regions involved in epileptogenesis that may not manifest observable clinical signs (14, 27, 36, 50–52) and may not be detectable through surface recording EEG electrodes (53, 54). This was evident in the cases of patients 1 and 2, where rs-fMRI signals were less affected by spatial resolution limitations. These identified networks meet the criteria for rs-SOZ, previously seen in children with drug-resistant epilepsy, as they were primarily located in gray matter regions and did not correspond to typical resting-state networks or spatial and temporal noise distributions (11). Similar to the resolution of rs-SOZ with therapy in drug-resistant epilepsy, both patient 1 and possibly patient 2 demonstrated the normalization of these rs-fMRI findings following antiseizure medication (27). This suggests that rs-fMRI may be able to differentiate between severe irreversible findings, such as those shown in patient 3, and those that may be treatable and transient conditions before progressing to irreversible chronic conditions such as chronic disorders of consciousness or epilepsy.

The current standard of care for suspected non-convulsive EEG-negative status epilepticus is empirical treatment based on the provider's judgment. The low-risk antiseizure medication is favored due to the risk–benefit ratio and the potential for restoring consciousness, given the relatively high mortality rate associated with disorders of consciousness (DoC). However, there are no clear guidelines for therapy children with SABI and suspected EEG-negative seizures. In this scenario, there is no known marker of response other than the clinical examination, which lacks a definitive timeframe or guidelines for treatment escalation. Our study demonstrates a biomarker that may show improvement in brain network function prior to improvement in the clinical examination, which can have implications for decisions regarding WLST in DoC.

### Limitations and further study

The decision to implement a new non-invasive diagnostic tool or to continue empirical treatment, both of which may or may not benefit the patient, ultimately rests with the treatment team's judgment. The process of increasing the level of evidence for the biomarker requires well-designed future research.

There are limitations and areas for further study. Exploring the interaction or association between EEG and rs-fMRI data may provide additional insights. Although EEG-based markers of covert consciousness are not currently clinically available at our institution, analyzing the passive EEG signal alone could offer valuable information on natural environmental stimuli and basal background activity, particularly during sleep, which has predictive value.

Rigorous research-based validation of network biomarkers in DoC is necessary to establish confidence in their clinical use. Encouragingly, the scientific community is actively engaged in conducting independent prospective studies, which supports the need for method validation. A prospective research protocol would be the optimal approach to advance this field. One proposed research design to validate the imaging biomarker is through the blinded application, where antiseizure medication is sequentially introduced and stopped to observe changes in rs-fMRI and task-fMRI, specifically focusing on spontaneous correlations and their behavioral correspondence. This can be achieved through the implementation of a single-case experimental design protocol (55).

Furthermore, it is important to note that long-term medication administration without a clear diagnosis is generally not recommended. However, in the context of acute coma, the administration of antiseizure medication is within the scope of practice and is justified due to the life-threatening nature of the situation. Furthermore, it may improve the patient's outcome. A well-designed research protocol would enable the differentiation of rs-SOZ in relation to the suppression of consciousness, epileptogenic activity, and/or seizures. By conducting rigorous research with appropriate protocols, we can further understand the role of the proposed imaging biomarker and its impact on the management and treatment of DoC.

### Patient and family perspectives

The perspectives of patients and their families are invaluable in understanding the impact of these imaging techniques on their experiences. Patient 1 and his parents provided their perspective through a live video interview conducted during the NCSs' World Coma Day event (1). They emphasize the importance of advanced neuroimaging in children with acute coma and advocate for increased awareness among the healthcare community regarding the possibility of positive outcomes even when the clinical examination appears poor. Patient 2 and his mother also shared their experiences through an audio-recorded interview for the same event (2). Patient 2 recalls his awareness during his ICU stay when his clinical examination indicated suppressed consciousness, and his mother discusses the challenges of prognostic uncertainty and the need for compassion and understanding from the care team. Patient 3's mother expressed the difficulty of not having a conclusive understanding of what caused her son's demise and hopes that this publication will inspire the healthcare community to advance the science to find answers for others in similar situations.

## Conclusion

The serial use of multimodal brain network evaluations, particularly rs-fMRI, holds promise in guiding neurointensivists to detect early pathological changes and implement more effective neuroprotective interventions in a timely manner. Patient 1's case highlights the advantage of serial assessments as the normalization of resting-state networks preceded improvement in the clinical examination, indicating the potential for anticipated recovery. These concepts may be applied to serial assessments in disorders of consciousness during acute illnesses to accurately predict reversibility or permanence, considering the risk of secondary injury mechanisms.

In these cases, rs-fMRI shows promise in discriminating along the spectrum of disorders of consciousness, ranging from brain death to recovery, aligning with bedside examinations, and improving outcome prediction. This finding is consistent with previous studies (41, 43, 44, 56–59).

Our case series presents the first reported case of a child with covert consciousness identified during acute disorders of consciousness, as well as the first pediatric patient with fMRI findings prior to brain death. The severity of connectivity abnormalities observed in these cases correlated with the outcomes. Furthermore, we reaffirm our previously reported biomarker of epileptogenesis in acute brain injury—disorders of consciousness, which can be detected using rs-fMRI (36).

Overall, our findings suggest that serial multimodal brain network evaluations in pediatric acute brain injury—disorders of consciousness, have the potential to provide early visualization of network disorder in the context of the resting-state networks, treatment effects, potential for ongoing covert consciousness, and aid in predicting outcomes. Continued research and application of these techniques could significantly impact the management and prognosis of patients with these conditions.

## Data availability statement

The original contributions presented in the study are included in the article/Supplementary material, further inquiries can be directed to the corresponding author.

## Ethics statement

Ethical review and approval was not required for the study on human participants in accordance with the local legislation and institutional requirements. Written informed consent from the patients/participants or patients/participants' legal guardian/next of kin was not required to participate in this study in accordance with the national legislation and the institutional requirements. Written informed consent was obtained from the minor(s)' legal guardian/next of kin for the publication of any potentially identifiable images or data included in this article.

## Author contributions

VB designed, led the study, and assisted with data analysis. BS interpreted the task-fMRI, drafting of methods, and interpretation. EG-C assisted with interpretation and drafting. KG assisted with figures and editing. MS assisted with drafting, editing, and mentorship. All authors contributed to the article and approved the submitted version.
