# Supplementary material for: Treatable brain network biomarkers in children in coma using task and resting-state functional MRI: a case series
Source: Front Neurol. 2023 Aug 10;14:1227195. doi: 10.3389/fneur.2023.1227195 (PMC10448513; doi:10.3389/fneur.2023.1227195)
Supplement: Supplementary file 1 [file Data_Sheet_1.pdf]

SUPPLEMENTARY MATERIAL

METHODS

EEG and fMRI: EEG-based assessments, task-fMRI and rs-fMRI sequences parameters, equipment, analysis, and interpretation procedures were performed as reported in our prior work,(1-3) with exceptions to the task-based task-fMRI procedures described below. The rs-fMRI was analyzed in a data-driven whole-brain manner (independent component analysis; ICA) to create the whole brain network profile, as demonstrated in a recent meta-analysis to demonstrate ability to localize seizure onset zones in adults and children.(4)

Task-fMRI: Task-fMRI requires 15-30 minutes, depending on the number of tasks performed. The staff training requirements are above those provided in MRI technician training. At PCH, in patient 1, the task-fMRI was performed by a cognitive neuroscientist and research psychologist with extensive experience in designing and performing task-fMRI language tasks in children and adults (BLS), under the supervision of the attending neurologist with fMRI expertise (VLB). The order of tasks was selected to place the most cognitively challenging task first to minimize fatigue.

The task-fMRI DOC-arousal protocol was initiated by stating the patient's first name preceding all paradigm and scanner-related instructions and motor commands, using the patient's primary spoken language(5). Developmentally-tailored tasks adapted from prior work in adults(6) were implemented. Patients with no history of following commands are not candidates for active task-based command following fMRI. However passive stimuli, such as flashing lights, are still applicable. Instructions and verbal stimuli were delivered using the most frequently spoken language (English). Auditory stimuli and instructions were presented through MR-compatible headphones. Visual stimuli were projected onto a mirror mounted on the head coil, with eyelids taped open by staff during this scan.

Passive visual stimulation and finger tapping, active arm motor command, with at least one language-related task was delivered in each session. Other tasks were completed as appropriate for the patient. Children unable to follow commands at baseline are not thought to be candidates for active task-fMRI, but passive tasks may still be clinically applicable.(7, 8)

ADDITIONAL PATIENT DETAILS

Patient 1 Initial clinical interventions: head of bed elevated, normoglycemia, goal sodium > 140 mmol/L, magnesium > 2 mmol/L, partial pressure of carbon dioxide 35-40 mmHg, oxygen saturation near 100%, hemoglobin > 8 mg/dL, temperature goal 36 – 37ºC.

Patient 2 Noting at D50 he possibly followed his first clear command, whereas other prior movements (hand grip, facial grimace, tongue movements) initially thought to be in response to command were later doubted by some providers as more likely due to stereotypical nature and observed response to environment noise or passive movement or touch of limbs (see supplemental material for day-by-day exam and medications, especially physical medicine and rehabilitation physician note on day 26).

Table S1. Patient 2 Daily Summary of Exam, Examiner Specialty, Sedating, and neuroactive medications

NS-1-5: Neurosurgeons
ICU-1: ICU provider
MED-1-3: Hospitalist/ICU provider
PMR-1: Physical Medicine and Rehabilitation Physician
PT-1: Physical Therapist
Disorder of Consciousness (DOC) Operational Definitions

- Coma: eyes closed, not clearly withdrawing (must be abduction to pain – flexion can be triple flexion (reflex) versus withdrawal (purposeful))
- UWS: unresponsive wakefulness syndrome, means eyes open but not clearly following a command
- MCS: minimally conscious syndrome, means eyes open and intermittently follows some command such as squeeze hand or move mouth
- Conscious – awake and consistently following commands

Day	examiner	**Medications,** as noted in neurosurgery notes. Medications in manuscript verified from medical record medication administration records directly.	**Exam quotes** from neurosurgery, who were the first to examine and intervene, preferentially quoted here and if exam not available then other medical staff's exam (see examiner column). Manuscript PEDS-GCS score preferentially from neurocritical care provider.	DOC – catatorized from data from exminer's note by research staff
1	NS-1	PRN rocuronium 60 mg IV q1<br>PRN morphine 3 mg IV q2	Posterior craniectomy, "sudden onset of headache, **rapid loss of consciousness** with a 7 cm posterior fossa hematoma with brainstem compression, hypertension and relative bradycardia taken emergently for EVD placement and posterior fossa decompression (craniectomy) and expansion duraplasty. taken to an outside ER where his **GCS was reported a 7.** He was intubated. CT head obtained with continued posterior hematoma, cerebellar edema, intraventricular hemorrhage, hydrocephalus, brainstem compression. ventricular tachycardia, right pupil was noted to be dilating	coma
2	NS-1	Dexmedetomidine 0.2 mcg/kg/hour IV<br>PRN fentanyl 60 mcg q1<br>PRN rocuronium 60 mg IV q1<br>PRN morphine 3 mg IV q2	Intubated/Sedated. Pupils 3mm reactive bilaterally.	Coma
3	NS-1	Dexmedetomidine 0.2 mcg/kg/hour IV<br>Fentanyl 0.5 mcg/kg/hour of 50 mcg/mL<br>PRN fentanyl 60 mcg q1<br>PRN rocuronium 60 mg IV q1	Intubated/Sedated. Pupils 3mm reactive bilaterally.	Coma
4	NS-1	Dexmedetomidine 0.2 mcg/kg/hour IV<br>Fentanyl 0.5 mcg/kg/hour of 50 mcg/mL	**GCS: E1M1V1**. Intubated/lightly sedated. Pupils 3mm reactive bilaterally. +corneals	Coma
5	NS-1	Dexmedetomidine 0.4 mcg/kg/hour IV<br>Fentanyl 0.5 mcg/kg/hour of 50 mcg/mL	**GCS: E1M1VT.** Intubated/sedated. Pupils 3mm reactive bilaterally. +corneals	Coma
6	NS-2	Dexmedetomidine 0.4 mcg/kg/hour IV<br>Fentanyl 0.5 mcg/kg/hour of 50 mcg/mL	Still on DEX and Fent: Intubated/sedated. B/l LE flexor response, minimal finger movement on deep stimuli on L UE. No movement noted on Rt UE, Pupils 3mm reactive bilaterally. +corneals	Coma vs UWS
7	NS-3	Dexmedetomidine 0.4 mcg/kg/hour IV<br>Fentanyl 0.5 mcg/kg/hour of 50 mcg/mL	Intubated/sedated. opens eyes to stimuli/call, B/l flexor response of all 4 extremities, Pupils 3mm reactive bilaterally. +corneals	UWS

8	NS-1	Dexmedetomidine 0.1 mcg/kg/hour IV	Intubated/sedated. **Occasionally opens eyes to voice**, pupils reactive	UWS
9	NS-1	OFF continuous SEDATION, rare occasional prn	Intubated/sedated. Occasionally opens eyes to voice, pupils reactive. **Squeezes hands to commands (?),** minimal movement bilateral LE	Initially thought might be MCS-; but this movement found to be reflexive, thus UWS
10	NS-1	1 dose morphine 60 mcg IV	Intubated/ minimally sedated. Occasionally opens eyes to voice, pupils reactive. **Squeezes hands stereotypes to command and also nonspecifically, indeterminate significance**	UWS
11	NS-1	OFF continuous SEDATION, rare occasional prn	Intubated/ minimally sedated. Occasionally opens eyes to voice, pupils reactive. **Squeezes hands stereotypes to command and also nonspecifically, indeterminate significance** Flexes to painful stim.	UWS
12	NS-1	OFF continuous SEDATION, rare occasional prn	Intubated/ minimally sedated. Occasionally opens eyes to voice, pupils reactive. Flexes to painful stim.	UWS
13	NS-4	OFF continuous SEDATION, rare occasional prn	Intubated/ minimally sedated. Occasionally opens eyes to voice, pupils reactive. Flexes weakly to painful stim.	UWS
14	NS-4	OFF continuous SEDATION, rare occasional prn	Intubated/ minimally sedated. Occasionally opens eyes to voice, pupils reactive. Flexes weakly to painful stim.	UWS
15	NS-1	OFF continuous SEDATION, rare occasional prn	Intubated/ minimally sedated. Occasionally opens eyes to voice, pupils reactive. Flexes weakly to painful stim.	UWS
16	NS-3	OFF continuous SEDATION, rare occasional prn	Intubated/ minimally sedated. Occasionally opens eyes to voice, pupils reactive. Flexes weakly to painful stim.	UWS
17	NS-1	OFF continuous SEDATION, rare occasional prn	Intubated/ minimally sedated. Occasionally opens eyes to voice, pupils reactive. Flexes weakly to painful stim.	UWS
18	NS-4	OFF continuous SEDATION, rare occasional prn	minimally sedated. Occasionally opens eyes to voice, pupils reactive. Flexes weakly to painful stim.	UWS
18	ICU-1	OFF continuous SEDATION, rare occasional prn	Recommend discussion with palliative care and support decision making	
19	NS-2	OFF continuous SEDATION, rare occasional prn	minimally sedated. Occasionally opens eyes to voice, pupils reactive. Flexes weakly to painful stim	UWS
20	NS-2	OFF continuous SEDATION, rare occasional prn	minimally sedated. Occasionally opens eyes to voice, pupils reactive. Flexes weakly to painful stim	UWS
21	NS-2	OFF continuous SEDATION, rare occasional prn	minimally sedated. Occasionally opens eyes to voice, pupils reactive. Flexes weakly to painful stim	UWS
22	NS-1	OFF continuous SEDATION, rare occasional prn	Occasionally opens eyes to voice, pupils reactive. Flexes weakly to painful stim. **Left had contracts into grip but did not open to command.** **No commands on right or LE**.	UWS

23	NS-1	OFF continuous SEDATION, rare occasional prn	Occasionally opens eyes to voice, pupils reactive. Flexes weakly to painful stim. Left had contracts into grip but did not open to command. No commands on right or LE.	UWS
24	NS-1	OFF continuous SEDATION, rare occasional prn	Occasionally opens eyes to voice, pupils reactive. Flexes weakly to painful stim. **Possibly starting to follow commands by sticking out tongue (?)**.	UWS
25	NS-1	Zolpidum 5 mg	Occasionally opens eyes to voice, pupils reactive. Flexes weakly to painful stim. Sticking out tongue and other oral movements found to be stereotypes and happening relatively frequently to many types of stimulus, thus not command following	UWS
25	NS-5	Zolpidum 6 mg	Occasionally opens eyes to voice, pupils reactive. Flexes weakly to painful stim.	UWS
26	PMR-1	Zolpidum 5 mg	low level neuro status. **opens his eyes**, but **doesn't follow commands or initiate movement.** " Her Exam: NEURO: , no visual tracking, atypical grimace -like movement with his mouth/lips **:** **Intermittent lower face grimace/"angry face" , sometimes often triggered by manipulation or touch of a limb, or in response to a command.**	UWS
27	NS-5	Zolpidum 7 mg	Occasionally opens eyes to voice, pupils reactive. Flexes weakly to painful stim. Possibly starting to follow commands by sticking out tongue.	UWS
28	NS-5	Zolpidum 8 mg	**Occasionally opens eyes to voice,** pupils reactive. Flexes weakly to painful stim. **Possibly starting to follow commands** by sticking out tongue (not consistent).	UWS
29	NS-1	Zolpidum 9 mg	Occasionally opens eyes to voice, pupils reactive. Flexes weakly to painful stim.	UWS
30	NS-1	Zolpidum 9 mg	Occasionally opens eyes to voice, pupils reactive. Flexes weakly to painful stim.	UWS
31	NS-1	Zolpidum 10 mg	Occasionally opens eyes to voice, pupils reactive. Flexes weakly to painful stim.	UWS
32	NS-1	Zolpidum 11 mg; **keppra 60mg/kg**	Occasionally opens eyes to voice, pupils reactive. Flexes weakly to painful stim.	USW
33	NS-1; MED-1	Zolpidum 18 mg<br><br>**phenobarbital 20mg/kg with some ?improvement**<br><br>**keppra 20mg/kg divided BID x 3-4 weeks plan**	Occasionally opens eyes to voice, pupils reactive. Flexes weakly to painful stim.<br><br>**Glasgow coma scale: 8**<br>Neuro: asleep<br><br>**Zolpidum stopped due to lack of clear improvement in arousal and possibly worse as determined by the neurocritical care and intensivist teams together.**	UWS
33	MED-1			Coma to UWS
34	NS-1	Keppra 20 mg/kg divided bid	Occasionally opens eyes to voice, pupils reactive. Flexes weakly to painful stim.	UWS
35	NS-1	Keppra 20 mg/kg divided bid	Occasionally opens eyes to voice, pupils reactive. Flexes weakly to painful stim.	UWS
36	NS-1	Keppra 20 mg/kg divided bid	Occasionally opens eyes to voice, pupils reactive. Flexes weakly to painful stim.	UWS
37	NS-1	Keppra 20 mg/kg divided bid	Occasionally opens eyes to voice, pupils reactive. Flexes weakly to painful stim.	UWS
38	NS-1	Keppra 20 mg/kg divided bid	Occasionally opens eyes to voice, pupils reactive. Flexes weakly to painful stim.	UWS
39	NS-1	Keppra 20 mg/kg divided bid	Occasionally opens eyes to voice, pupils reactive. Flexes weakly to painful stim.	UWS

40	NS-1	Keppra 20 mg/kg divided bid	Occasionally opens eyes to voice, pupils reactive. Flexes weakly to painful stim.	UWS
41	NS-4	Keppra 20 mg/kg divided bid	Occasionally opens eyes to voice, pupils reactive. Flexes weakly to painful stim.	UWS
42	NS-4	Keppra 20 mg/kg divided bid	Opens eyes spontaneously during day hours, pupils reactive. Flexes weakly to painful stim.	UWS
43	NS-4	Keppra 20 mg/kg divided bid	Opens eyes spontaneously during day hours, pupils reactive. Flexes weakly to painful stim.	UWS
44	NS-1	Keppra 20 mg/kg divided bid	Opens eyes spontaneously **during day hours**, pupils reactive. Flexes weakly to painful stim.	UWS
45	NS-1	Keppra 20 mg/kg divided bid	Opens eyes spontaneously **during day hours**, pupils reactive. Flexes weakly to painful stim.	UWS
46	NS-1	Dexmedetomidine 0.4 mcg/kg/hour IV Keppra 20 mg/kg divided bid	Opens eyes spontaneously during day hours, pupils reactive. Flexes weakly to painful stim.	UWS
47	NS-1	Dexmedetomidine 0.6 mcg/kg/hour IV Meperidine Injectable 3 mg IV Every 2 hours PRN once Keppra 20 mg/kg divided bid	Opens eyes spontaneously during day hours, pupils reactive. Flexes weakly to painful stim.	UWS
48	NS-4	Oxycodone 2.5 mg NG tube q4 prn Dexmedetomidine 0.8 mcg/kg/hour IV Fentanyl 50 MCG IV Every 1-hour PRN Keppra 20 mg/kg divided bid	Opens eyes spontaneously during day hours, pupils reactive. Flexes weakly to painful stim.	UWS
49	NS-4	Dexmedetomidine 0.8 mcg/kg/hour IV Fentanyl 50 MCG IV Every 1-hour PRN Keppra 20 mg/kg divided bid	Opens eyes spontaneously during day hours, pupils reactive. Flexes weakly to painful stim.	UWS
50	PT-1	Dexmedetomidine 0.8 mcg/kg/hour IV Fentanyl 50 MCG IV Every 1-hour PRN Oxycodone 2.5 mg NG tube q4 prn Keppra 20 mg/kg divided bid	Turns head side to side when asked (possibly first clear time of following a command?)	MCS
51		Oxycodone 2.5 mg NG tube q4 prn Keppra 20 mg/kg divided bid		
52		Keppra 20 mg/kg divided bid		

53	PT-2	Keppra 20 mg/kg divided bid	Able to sit strapped in wheelchair with full support of head and body	MCS
54		Keppra 20 mg/kg divided bid	Transfer to re-hab unit	
55	MED-2	Keppra 20 mg/kg divided bid	NAD, non-toxic, **no major responses to stimulation, seems awake with eyes open;** no verbalization, increased tone	UWS vs MCS
56	MED-2	Keppra 20 mg/kg divided bid	NAD, non-toxic, no major responses to stimulation**, seems asleep**; no verbalization, increased tone	UWV vs MCS
57	MED-1	Keppra 20 mg/kg divided bid	NAD, non-toxic, appears alert; no verbalization, increased tone, **moves head laterally, on cue, intermittently**	MCS+
58	MED-1	Keppra 20 mg/kg divided bid	NAD, non-toxic, appears alert; NAD, non-toxic, appears alert	UWS
59	MED-1	Keppra 20 mg/kg divided bid	NAD, non-toxic, appears alert; no verbalization, increased tone, **moves head laterally, on cue, intermittently**	MCS+
60	MED-1	Keppra 20 mg/kg divided bid	NAD, non-toxic, appears alert; no verbalization, increased tone, moves head laterally, on cue, intermittently	MCS+
61	MED-1	Keppra 20 mg/kg divided bid	NAD, non-toxic, appears alert; no verbalization, increased tone, moves head laterally, on cue, intermittently	MCS+
62	MED-1	Keppra 20 mg/kg divided bid	NAD, non-toxic, alert but nonverbal, no agitation; no verbalization, increased tone, moves head laterally, on cue, intermittently	MCS+
63	MED-1	Keppra 20 mg/kg divided bid	NAD, non-toxic, alert but nonverbal, no agitation; no verbalization, increased tone, moves head laterally, on cue, intermittently	MCS+
64	MED-3	Keppra 20 mg/kg divided bid	NAD, non-toxic, alert but nonverbal, no agitation, sitting in shower chair; no verbalization, increased tone, moves head laterally, on cue, intermittently	MCS+
65	NS-1	Keppra 20 mg/kg divided bid	no verbalization, increased tone, **responds in a delayed fashion to questions with a head nod**	**Conscious**

Patient 3 Given outcome of brain death highly detailed additional information provided to evaluate for confounders herein. Positive infant death risk factors: white male, twin-gestation, 3 weeks early – 37 weeks gestation, and not sleeping on his back.

Maternal/placental/fetal triad data, including preconception status: mother reports receiving prenatal care throughout, with several normal ultrasounds. In the last week of pregnancy, she had proteinuria, but no elevation in blood pressure, nor seizure. Had spontaneous labor, then cesarian section with no complications. Mom was 42 years, and dad 39 at conception.

Maternal birth history: 8 pregnancies, 1 spontaneous miscarriage in first trimester, 1 abortion.

PMHx: patient's birth weight was 7 lbs, 13 oz, 53 cm length; and twin was 6 lbs, 12 oz; both breast fed with supplemental formula; no feeding issues; several scheduled well visits to pediatrician unremarkable; circumcision without complication. State mandated screens negative. No genetic testing.

Family Hx: Mother was a smoker prior to the pregnancy. Father had adult onset insulin dependent diabetes. Mother report a history of dad having delusions, but neither she nor he have had any psychiatric formal diagnoses. Mother denied emotional/mental related symptoms. Paternal grandfather with major depressive disorder. Mothers first child was born when she was 15 years old. She has 6 children who are alive, age 2 mo – 24 years, are heathy, with no developmental, behavioral, educational issues

Education: mother and father finished high school in regular classes without extra help. Mother had some college.

Vitals: 4.98 kg, 58 cm height, head circumference 38.5 cm, temperature 32 °C on arrival then averaging 37 °C afterwards, HR 120-210 bmp, RR 30s, mean blood pressures 49-96 with average in 60-70 range, urine output 1.7 cc/kg/hour average, and cerebral oximetry was 94% on day 4. **Ventilator Settings:** servo mode, SIMV, PRVC, PS, rate 26-30 (decreased to 5 only for BD apnea test), FIO2 40, tidal volume 35 mL, PEEP 5 cm H20, pressure support 8 cm H20, peak inspiratory pressure 22 cm H20. **Medications:** Day 1, epinephrine iv, norepinephrine bitartrate iv then discontinued. Day 4, fentanyl 20 mcg iv once, ampicillin-sulbactam 220 mg iv, furosemide 4 mg iv once.

Exams with reported relevant unique or validating findings between providers and over time: **Ophthalmology:** Day 1, No response to light OU, external exam quiet with normal cornea and conjunctiva, optic nerve with normal cup-to-disk ratio 0.05 bilaterally, normal retina, vasculature, and macula, no papilledema, and no retinal hemorrhage. **Neurosurgery, Trauma Service**: Day 1, Glasgow coma scale (GCS) 3, anterior fontanelle open and soft, exams without evidence of trauma. **Intensivist:** Day 1-3, GCS 3, unresponsive to stimulation, no movement, no brainstem reflexes. Day 2-4, persistent agonal breathing at RR 8 bpm. Day 4-5, cessation of agonal breathing. ***Day 5, complete brain death exam including apnea test with preoxygenation to PaO2 > 200 mm Hg was without respiratory effort and showed rise in PaCO2 > 20 mm Hg, and confirmation by EEG with electrocerebral silence. Thus, he was declared brain dead.(9, 10)*** The brain death exams: pupils were 4 mm and fixed to light, absence of ocular movement to oculocephalic testing. There was absence of: corneal reflexes, facial movement to noxious stimuli, pharyngeal-gag, and tracheal-cough response, and oculocephalic and oculovestibular reflexes. **Neurology:** Day 1, non-dysmorphic, fontanelle flat, normocephalic, no neurocutaneous stigmata, no brainstem reflexes, no movement, flaccid deep tendon reflexes, and flaccid tone. Day 2, unchanged except for ***spontaneous movement of right hand/wrist, with external rotation and extension intermittently without clear provocation, no reliable reproduction of movement to noxious stimuli.*** Day 3-4, fontanelle bulging, agonal breathing above the ventilator, no movements, mild to moderate generalize muscle tone. Day 5, exam consistent with brain death.

Laboratory: Day 1, initial pH was 6.88, lactic acid 9.9 mmol/L, glucose 276 mmol/L, and bicarbonate < 10 mmol/L. The metabolic panel showed (in units of mmol/L unless specified) sodium 145, potassium 4.6, chloride 119 (mild elevation), CO2 18 mmHg, anion gap 8, glucose 136 (mild elevation), blood urea nitrogen 20 mg/dL, creatinine 0.41, BUN/Cr ratio 49 (10-28 normal), and calcium 8.3 mg/gL. Ammonia was 67 (11-50 umol/L), amylase 9 (1-50 U/L), lipase 19 (10-53 U/L), procalcitonin 23.62 – elevated (0.00-0.05 ng/mL), aspartate and alanine aminotransferase 162 and 253 IU/L, total bilirubin 0.3 mg/dL, alkaline phosphatase 253 U/L, albumin 2.2 g/dL, and total protein 3.8 g/dL. His serum complete blood count on day 4 of admission showed hemoglobin of 9.6 gm/dL (normal), white blood cell count of 6.0 k/uL (normal), platelet count of 33 K/uL (low), red blood cell morphology of burr cells, fragments, and polychromasia (mild abnormalities). His PT was 19.8 sec, INR 1.7, APTT of 37.2 sec (mild elevation). D-Dimer was 7.8 ug/ml FEU (marked elevation). Tracheal cultures reveal contaminates of Staphylococcus aureus and Klebsiella oxytoca and negative viral panel. Urine and blood cultures were negative. Arterial blood gas on Day 5 showed pH 7.41, partial pressure of CO2 29 and O2 78 mmHg, base deficit 6.0, oxygen saturation 98%, oxyhemoglobin 2.0%, methemoglobin 1.2%, and deoxyhemoglobin 2.0%. Day 1 urine analysis was unremarkable except with 3+ of glucose, protein, hemoglobin, and ascorbate (which can cause false positive results for urine containing blood). **Toxicology:** Day 1, toxicological evaluation of urine gas chromatography-mass spectrometry was positive for amphetamine, methamphetamine, and niacinamide, which is reported to be a 70% match compared to internally developed spectral libraries by Sonora Quest, which is an unconfirmed medical screen, and is not approved by the FDA, and is regulated under the Clinical Laboratory Improvement Amendments as qualified to perform high-complexity testing for clinical purposes, and is not investigational or for research. However, ***confirmatory tests were negative*** by quantitative liquid chromatography-tandem mass spectroscopy amphetamine ≤ 50ng/mL (positive cutoff is 200 ng/mL), and MDA, MDEA, MDMA, methamphetamine, and phentermine were < 200 mg/mL. Fentanyl urine screen was negative.

Electroencephalogram: Flat and featureless, no evidence of cerebral activity or seizures at any point during recording. ***Electrocerebral silence*** by video monitored electroencephalogram according to ACNS guidelines, 18 channel EEG with ECG and pulse oximeter monitors, with testing for reactivity to exogenous stimuli on Day 5.

Body Imaging: CT of the abdomen and pelvis with contrast was consistent for findings suggesting shock (interloop fluid and ascites with no free air, fluid filled and hyper-enhancing bowel wall) and no evidence for solid organ injury. Chest x-rays over the course were consistent with improving atelectasis, underlying edema, and resolved left pneumothorax. Renal ultrasound demonstrated increased cortical echogenicity of uncertain significance, either due to age or medical renal disease.

Central Nervous System Imaging: Day 1, CT of the head showed a nondisplaced midline occipital bone fracture, and nonspecific scattered areas of hypoattenuations involving the periventricular white matter and basal ganglia, concerning for hypoxic injury. CT of the cervical spine was normal. Day 3-4, anatomical MRI showed initially more focal injury that progressed by the next day to total brain infarction. Day 5, transcranial doppler ultrasound (TCD) showed reversal of flow during diastole of bilateral middle cerebral arteries, consistent with markedly high increased intracranial pressure, and seen in adults with brain death.(11) Day 4, whole brain rs-fMRI was acquired and analyzed. It showed no detectable local or long-range neuronal networks and all the independent components detected were consistent with sources of noise as previously reported.(1, 2, 12)

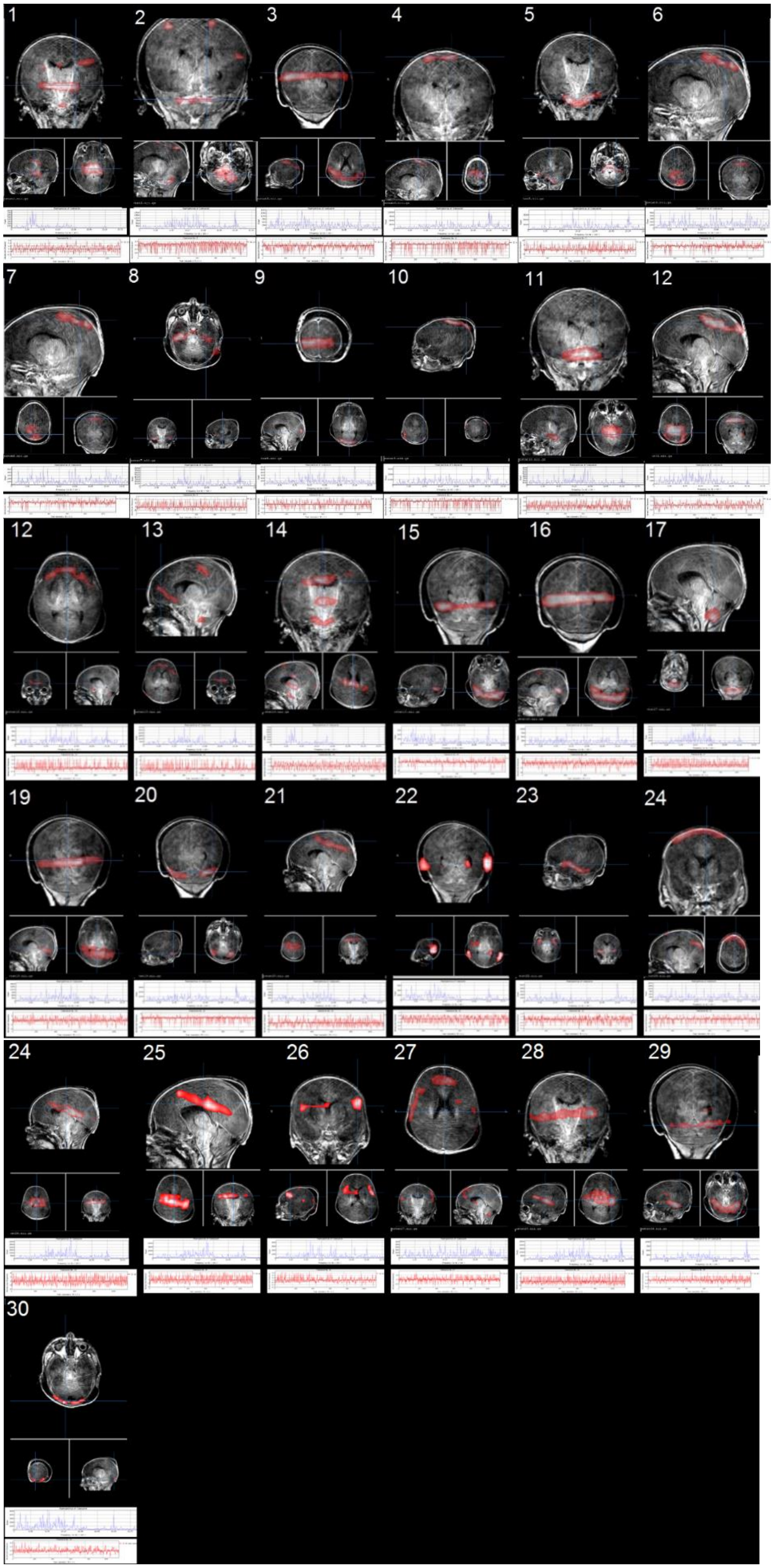
1
2
3
4
5
6
7
8
9
10
11
12
12
13
14
15
16
17
19
20
21
22
23
24
24
25
26
27
28
29
30

S-Figures 1. Patient 3: Resting State fMRI Independent Component Network Results for the patient's IC 1-30. Thumbnail views of all resulting independent components (IC) are shown, each with a single IC in the axial, coronal, and sagittal views. Each IC's corresponding time course of blood oxygen dependent (BOLD) are shown in the RED graph with normalized BOLD on y-axis and time over 10 minutes on the x-axis. Also, each IC's corresponding power spectrum of time course frequencies are shown in the BLUE graph with power on the y-axis and frequency in Hz/100 on the x-axis. The results are consistent with artifactual and physiological noise with no IC spatially conforming to any known brain network or atypical brain network. The time courses and power spectrums demonstrate highly regular generally fast signal compared to normal brain networks by rs-fMRI.

1. Boerwinkle VL, Cediel EG, Mirea L, Williams K, Kerrigan JF, Lam S, et al. Network Targeted Approach and Postoperative Resting State Functional MRI are Associated with Seizure Outcome. Ann Neurol. 2019:344-56.
2. Boerwinkle VL, Mohanty D, Foldes ST, Guffey D, Minard CG, Vedantam A, et al. Correlating Resting-State Functional Magnetic Resonance Imaging Connectivity by Independent Component Analysis-Based Epileptogenic Zones with Intracranial Electroencephalogram Localized Seizure Onset Zones and Surgical Outcomes in Prospective Pediatric Intractable Epilepsy Study. Brain connectivity. 2017;7(7):424-42.
3. Desai VR, Vedantam A, Lam SK, Mirea L, Foldes ST, Curry DJ, et al. Language lateralization with resting-state and task-based functional MRI in pediatric epilepsy. J Neurosurg Pediatr. 2018;23(2):171-7.
4. Chakraborty AR, Almeida NC, Prather KY, O'Neal CM, Wells AA, Chen S, et al. Resting-state functional magnetic resonance imaging with independent component analysis for presurgical seizure onset zone localization: A systematic review and meta-analysis. Epilepsia. 2020.
5. Del Giudice R, Blume C, Wislowska M, Lechinger J, Heib DPJ, Pichler G, et al. Can self-relevant stimuli help assessing patients with disorders of consciousness? Conscious Cogn. 2016;44:51-60.
6. Owen AM, Coleman MR, Boly M, Davis MH, Laureys S, Pickard JD. Using functional magnetic resonance imaging to detect covert awareness in the vegetative state. Arch Neurol. 2007;64(8):1098-102.
7. Perani D, Saccuman MC, Scifo P, Anwander A, Spada D, Baldoli C, et al. Neural language networks at birth. Proc Natl Acad Sci U S A. 2011;108(38):16056-61.
8. Shultz S, Vouloumanos A, Bennett RH, Pelphrey K. Neural specialization for speech in the first months of life. Dev Sci. 2014;17(5):766-74.
9. Nakagawa TA, Ashwal S, Mathur M, Mysore M. Clinical report—Guidelines for the determination of brain death in infants and children: an update of the 1987 task force recommendations. Pediatrics. 2011;128(3):e720-40.
10. Nakagawa TA, Ashwal S, Mathur M, Mysore M, Committee For Determination Of Brain Death In Infants C. Guidelines for the determination of brain death in infants and children: an update of the 1987 task force recommendations-executive summary. Ann Neurol. 2012;71(4):573-85.
11. Bathala L, Mehndiratta MM, Sharma VK. Transcranial doppler: Technique and common findings (Part 1). Ann Indian Acad Neurol. 2013;16(2):174-9.
12. Griffanti L, Douaud G, Bijsterbosch J, Evangelisti S, Alfaro-Almagro F, Glasser MF, et al. Hand classification of fMRI ICA noise components. NeuroImage. 2017;154:188-205.
